# Research of Recognition Method of Discrete Wavelet Feature Extraction and PNN Classification of Rats FT-IR Pancreatic Cancer Data

**DOI:** 10.1155/2014/564801

**Published:** 2014-12-04

**Authors:** Chayan Wan, Wenqing Cao, Cungui Cheng

**Affiliations:** ^1^College of Chemistry and Life Science, Zhejiang Normal University, Jinhua 321004, China; ^2^Shandong Exit-Entry Inspection and Quarantine Technology Center, Qingdao 266002, China

## Abstract

Sprague-Dawley (SD) rats' normal and abnormal pancreatic tissues are determined directly by attenuated total reflectance Fourier transform infrared (ATR-FT-IR) spectroscopy method. In order to diagnose earlier stage of SD rats pancreatic cancer rate with FT-IR, a novel method of extraction of FT-IR feature using discrete wavelet transformation (DWT) analysis and classification with the probability neural network (PNN) was developed. The differences between normal pancreatic and abnormal samples were identified by PNN based on the indices of 4 feature variants. When error goal was 0.01, the total correct rates of pancreatic early carcinoma and advanced carcinoma were 98% and 100%, respectively. It was practical to apply PNN on the basis of ATR-FT-IR to identify abnormal tissues. The research result shows the feasibility of establishing the models with FT-IR-DWT-PNN method to identify normal pancreatic tissues, early carcinoma tissues, and advanced carcinoma tissues.

## 1. Introduction

Pancreatic cancer is one of the most malignant tumors, for which it is extremely difficult to make an early diagnosis and surgical resection. And it is also difficult to get the different stages of fresh clinical pancreatic cancer specimens, so the early appearance of pancreatic cancer cannot be such easy to diagnose. The histological examination of specimens obtained by endoscopy method not only takes several days for diagnosis but also increases medical expenses. Therefore, it seems important to find a fast and simple way to diagnose the human pancreatic cancer [1].

The Fourier transform infrared (FT-IR) spectroscopy can provide every aspect of compositional system materials. Among the different spectroscopic methods that have been evaluated for utilization in distinguishing between normal and neoplastic tissues, FT-IR spectroscopy, as an advanced and promising technique, has shown its huge potential to this area. This FT-IR technique has become a reality with a large amount of information accumulated from clinical studies, trials, and FT-IR spectroscopy instruments developments. Such technique, being reagent-free, can rapidly and noninvasively detect changes in the biochemical composition of cells and tissues (at the molecular level), especially during carcinogenesis [2]. Some articles have been published about the application of FT-IR spectroscopy in the diagnosis of various human cancer cells so far [3–8].

But, in some normal conditions, FT-IR analysis cannot be such quickly and accurately used to obtain the exact results information. And how to use chemometrics to make the determination and classification of these complex systems change possibly, fast, and accurately has become a hot research in the instrument analysis [9–12].

Besides, wavelet transform (WT) is a much more effective signal processing method than the inheritance of the Fourier transform one and also plays an important role in signal analysis and feature extraction. In the multiresolution wavelet analysis, the results of the transformation (a wavelet factor) contain more valuable information, and the WT coefficients of each level are different for the same characterization of the signal. Therefore the wavelet decomposition coefficients may be considered as the characteristics of the signal, only with a small number of factors can be passed on the absorption spectra for a good reflection, and will be one of the more effective chemical analysis methods. Artificial neural networks (ANN) have a higher intelligence and have been widely used. Its calculation is simple and the prediction is accurate for the nonlinear issues [9, 10].

Our group has previously reported that normal and abnormal human lung tissues can be fast distinguished using CWT-HATR-FT-IR combined with support vector machine [9]. In this paper, we focus on the classification of normal and abnormal rats pancreatic tissues. The FT-IR is obtained by ATR-FTIR technique. The feature vectors, which represent spectral characteristics of the FT-IR, are extracted by using DWT multiresolution analysis methods. Probability neural network is used to classify the different pancreatic tissues.

## 2. Discrete Wavelet Transformation (DWT)

The discrete wavelet transformation (DWT) is the sum of the signal multiplied by the scaled and shifted mother wavelet function. The wavelet coefficients are a function of scale and position. Normally wavelet decomposition consists of calculating the “resemblance coefficients” between the signal and the wavelet located at position *b* of scale *a*. If the coefficients are large, the resemblance is strong. Otherwise it is weak. The coefficients *C*(*a*, *b*) are calculated as [13–15]

(1) Ca,b=∫Rst1aψt−badta∈R+−0, b∈R,

where *ψ* is wavelets, *s* is signal, *a* is scale, and *b* is position.

## 3. Probability Neural Network (PNN)

PNN is the feed-forward network model of artificial neural network based on the theory of statistics with Parzen window function as the activation function. PNN absorbs the advantages of radial basis function neural network (RBF-NN) [16] and classical theory of probability density estimation and compares with the traditional feed-forward neural network, which especially has the remarkable advantage in the pattern classification aspect.

**Figure 1:**
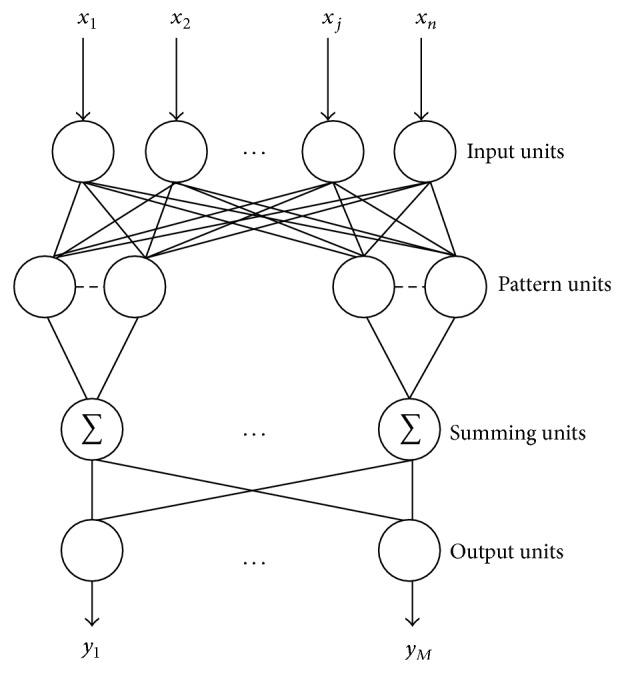
The topological structure of PNN.

The topological structure of PNN is shown in Figure 1. The first and last layout, respectively, denote input and output layout; the two middle layouts are hidden layouts. The first hidden layout is the pattern unit layout with Parzen window function as the activation function; the second one is summing unit layout which alternatively sums the output of the first one. The training methods of PNN mostly have recursion orthogonal least square algorithm (ROLS) and recursion least square (RLS). Both methods have quick convergence rate; in contrast, the training rate of the second one is quicker and has higher training precision.

To the input vector *x*, output value *Y*_*j*_ of the *i*th nerve cell in the output layout of PNN can be written as

(2) $$\begin{array}{c} Y_{j}=\overset{M}{\underset{k=1}{\sum }}w_{jk}H_{k}\left(x\right),\;j=1,2,\ldots ,M,\\ H_{k\left(x\right)}=\overset{n_{k}}{\underset{i=1}{\sum }}P_{i}\left(\left\|x-c_{kj}\right\|\right), \end{array}$$

where *x* is input vector with dimension; *H*_*k*_(*x*) is output of the *k*th unit in the second hidden layout; *w*_*jk*_ is connected weight between the *j*th nerve cell in the second hidden layout and the *k*th nerve cell of output layout; *P*(∗) is Parzen window function; *c*_*kj*_ is the *k*th center vector of the *j*th class in the first hidden layout; *n*_*k*_ is the number of the center vectors of the *k*th class in the first hidden layout; ‖·‖ is Euclidean norm; *M* is the number of nerve cells in the output layout.

## 4. Materials and Methods

### 4.1. Apparatus

ANicolet (Madison, WI, USA) NEXUS 670 FTIR spectrometer, equipped with a temperature-stabilized deuterated triglycine sulphate (DTGS) detector and diamond attenuation total reflection (ATR) accessory, was used. The spectral range is 4000–650 cm^−1^ with resolution 2 cm^−1^ and the cumulative number is scan of 64 times.

### 4.2. Chemicals and Reagents

Pentobarbital sodium and dimethylbenzanthracene (DMBA) were AP quality from Sinopharm, China. Tumor specific antigen (TSA) was for laboratory use only from Shanghai Huayi Bio Technology Co., Ltd., China.

### 4.3. Materials

6–8-week-old, 160 normal Sprague-Dawley (SD) rats, about 180 g, half male and half female, were used in the experiment. All rats were provided by the Department of Animals, Jinhua Institute for Drug Control, China, and were permitted by animal protection association. Then the SD rats were randomly divided into three groups A, B, and C: 70 rats for group A (DMBA group), another 70 rats for group B (DMBA + TSA group), and last 20 rats for group C (control group). None is fed but water for 24 h before the operation. 2% pentobarbital sodium (1.5 mL/kg) was injected into the SD rats' abdominal cavity where is about 1 cm incision into the upper middle abdomen for anesthesia after the exposure of the pancreas, the membrane and partial terms of pancreas were cut from the tail of body in deep 1 mm, group A and group B were put into 9 mg DMBA, and then the abdomen was stitched after stitching the membrane of pancreas. They were fed in the general environment with free drinking purified water and full price nutrition diet particles. Group B was weekly injected into the SD rats' abdominal cavity with 1 *μ*g/mL TSA 1 mL. In addition to natural death, group A (7, 10, and 20 SD rats) and group B (6, 10, and 20 SD rats) will be killed, respectively, in the third, fourth, and fifth month randomly; then all group C will be killed in the fifth month [1]. The anatomical pancreatic tissues were washed in physiology salt and then the samples were directly measured to collect their FTIR data in three hours; the specimens were taken at the same time for pathological analysis. The sample tissues included 220 pancreatic normal tissues, 120 early-staged cancerous tissues, and 100 advanced cancerous tissues.

### 4.4. Spectral Measurements

The FT-IR spectrum background was recorded before collecting the sample's FT-IR spectrum.Reference spectra were recorded using a blank diamond-ATR. Single beam spectra were obtained for all the samples and the ratio of the spectra against the background spectra of air was used to present the spectra in absorbance units. After each experiment the diamond-ATR was thoroughly washed with absolute ethyl alcohol and dried with nitrogen, and its spectra were examined to ensure that no residue from the previous experiment was retained on the diamond surface. All of the tissue samples were dried with absorbent cotton and further dried with nitrogen. All the experiments were repeated three times and the averaged spectra were used for further analysis.

### 4.5. Data Analysis

The FT-IR of samples was obtained by measurement. The absorption values from different wave bands based on the characters of the absorption value were obtained by copying data method. MATLAB software was used for wavelet transition analysis. Using Daubechies wavelet, which has a good detection capability of the signal singularity, as the analysis wavelet, one-dimensional DWT is done to the FT-IR spectra of samples under different scales. Five layers of the samples character variable were picked up by selecting a decomposition level whose difference degree was the most obvious. Through a comparative analysis, two layers (3 and 4) were selected to extract eigenvector. The number of training samples and testing samples is 230 and 210, respectively. The selected character variables were used for PNN training and identification.

## 5. Results and Discussion

### 5.1. FT-IR Analysis

**Figure 2:**
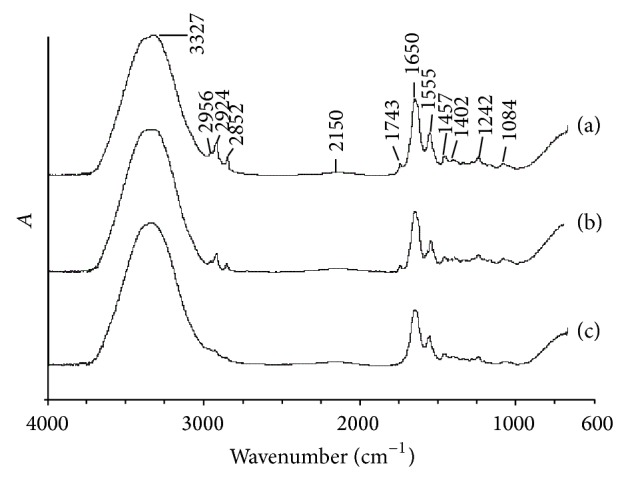
FT-IR spectra of pancreatic tissue samples. (a) Normal tissues; (b) early carcinoma tissues; (c) advanced carcinoma tissues.

Figure 2 shows the FT-IR spectra of pancreatic normal, early carcinoma, and advanced carcinoma tissues.

As shown in Figure 2, the location, intensity, and shape of the absorbance peak change with the normal, early carcinoma, and advanced carcinoma tissues. The hydroxyl absorption peak from protein, nucleic acid, and grease is located at 3327 cm^−1^ with a similar intensity. The pancreatic normal tissue has three obvious absorption bands, which were 2956, 2924, and 2852 cm^−1^, respectively, in the range of 3000~2800 cm^−1^ of saturated C–H bond stretching vibration region. But, with the development of canceration, these absorption peaks were weakened. The carbonyl absorption peak from ester of fat is located at 1743 cm^−1^, which is also weakened after canceration. It will disappear when it becomes advanced carcinoma. The carbonyl group absorption peak from protein is located at 1650 cm^−1^ (including water molecules in the hydroxyl in the absorption of 1640 cm^−1^), and its intensity decreases as the cancerous tissue progresses. Absorption peak from amide II band is located at 1555 cm^−1^, and its intensity also decreases as the cancerous tissue progresses. The other spectrum bands, such as symmetrical flexing vibration and asymmetrical flexing vibration of diphosphate ester from nucleic acid, had absorption peaks located at 1084 cm^−1^ and 1242 cm^−1^, respectively.

The FT-IR from the normal and early carcinoma tissues has very closed absorbance and it is difficult to distinguish between them only by experience. Sowe use one-dimensional discrete wavelet transformation to extract their features for further classification.

The higher information quantities exist in 3600~2800 cm^−1^ and 2000~650 cm^−1^. The region 3600~2800 cm^−1^ includes the absorbability of O–H, N–H stretching bands, C–H aliphatic asymmetric and symmetric stretching vibration band and the character is not obvious. The region 2000~650 cm^−1^ includes fingerprint region which contains more molecule structure information. Thus we use the region 2000~650 cm^−1^ for PNN training and identification.

### 5.2. DWT Analysis of FT-IR Data

Appropriate feature description is considered to be one of the most important components of classification procedures. The result of feature extraction is a more concise description that still retains most of the spectrum characteristics.

In this paper, we scale the FT-IR data to the range 4000~650 in order to perform analysis wavelet. All of the FT-IR spectra data contains a lot of redundant information. Extracting features from the FT-IR data can reduce the computational burden and improve results.

The proper wavelet base and scale should be determined by analyzing the signal spectra property and the comparison of decomposition results with different wavelet bases and decomposition levels [13]. During the multiresolution wavelet decomposition process, the suitable wavelet basis function and wavelet decomposition level are determined based on the comparison of the FT-IR signals and the signals' decomposition effects under different resolution. The standard selection is to extrude several characteristic peaks in the original spectra and select a good smoothing wavelet. After comparing Haar, Daubechies, Mexican hat, Meyer, Morlet, and Symlets wavelet decomposition, we choose Daubechies wavelet as “wavelet analysis.” Two characteristic peaks in the original FT-IR signal of wavelet domain are used for further extraction of its eigenvalues. The pancreatic normal, early carcinoma, and advanced carcinoma tissues were performed by discrete wavelet transformation separately; the decomposition level is set as 5. After analysis we choose the two levels 3 and 4 to extract the features.

**Figure 3:**
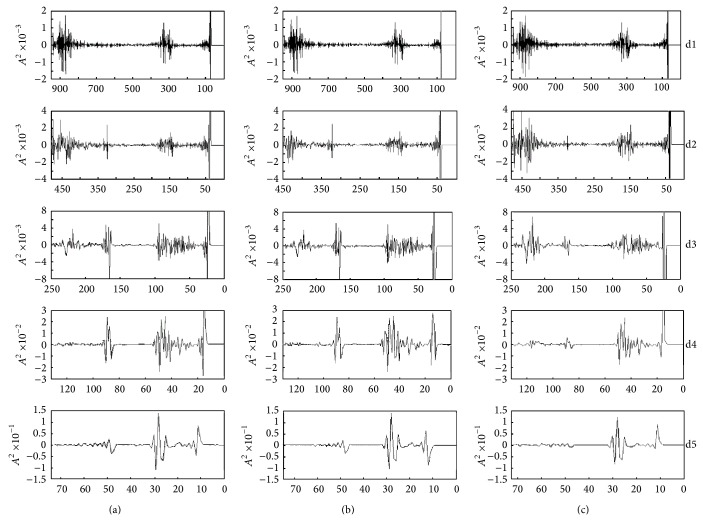
The result of multiresolution decomposition of normal, early carcinoma, and advanced carcinoma tissues' FTIR with discrete wavelet transformation. (a) Normal tissues; (b) early carcinoma tissues; (c) advanced carcinoma tissues.

Figure 3 shows preprocessed FT-IR spectra of normal, early carcinoma, and advanced carcinoma tissues' DWT coefficients, where d1–d5 indicates detailed information after decomposition (fine to coarse).

From Figure 3, we could see that it is difficult to discern the main features of its spectrum from the wavelet domain if the discrete wavelet transform resolution is fine because of too much detailed information. The detailed information is sensitive to spectrum change, has strong response to characteristic peak of the original various spectra, and is not good for the feature extraction.

**Figure 4:**
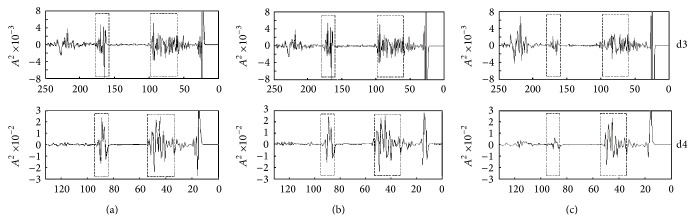
Division of feature region of high frequency components after discrete wavelet multiresolution decomposition. (a) Normal tissues; (b) early carcinoma tissues; (c) advanced carcinoma tissues.

In order to effectively extract representative characteristics within two scales of discrete wavelet, the spectra in each scale are divided into two representative regions, respectively. Various features are defined as energy of the decomposition in the DWT domain.  Figure 4 is the division diagram of the feature regions. Four feature regions of two details in the DWT domain, whose feature values are the spectra energy in the four feature regions, form the feature vector.

### 5.3. Identified PNN and Application of the Results

After testing, we define the structure of PNN as nine nodes in the input layer, four nodes in the hidden layer, and three nodes in the output layer; the error is 0.01.

The successful training network studied how to identify pancreatic normal, early carcinoma, and advanced carcinoma tissues. For the training process, we used nine input layer nodes of PNN structure, followed by normalized nine feature vectors. The output layer nodes were divided into category 1: normal tissues, category 2: early carcinoma tissues, and category 3: advanced carcinoma tissues. The trained network was used to verify the data of 440 different samples. The input data is the eigenvector extracted from the wavelet transform of the original FTIR. The results are showed in Table 1.

Based on the results in Table 1, basically, the three different types of samples (the pancreatic normal, early carcinoma, and advanced carcinoma tissues) are correctly identified. The eigenvectors of the different samples that were extracted from the wavelet transform of the FT-IR have significant difference so that the high accuracy of the classification can be achieved. It is 100% for identifying the pancreatic advanced carcinoma tissues because there is significant difference among them. It is difficult to distinguish early carcinoma tissues when canceration degree is not high. The recognition accuracy rate of normal and early carcinoma tissues is lower than advanced carcinoma tissues because the difference is very small.

## 6. Conclusion

It is easy to do the FT-IR measurement directly using diamond-ATR, which does not need to have the pretreated samples. There is a significant difference between normal pancreas tissue and advanced cancer, but to the early cancer the difference of their FT-IR spectra is not such obvious. Some important features were extracted to the FT-IR signals in the DWT domain, and probability artificial neural network pattern for recognition and classification based on their energy was also used. Network method for complex and ambiguous and cross information applications has special value and the use of radial basis function neural networks such as scientific evaluation system to avoid identification of the human experience and a number of other methods of complexity are simple, rapid, and accurate. Therefore, FT-IR through discrete waveletfeature extraction using probability neural network for early identification of pancreatic cancer has a more wide application prospect.

## Figures and Tables

**Table 1:** Recognition results of probability neural network.

	Normal tissues (%)	Early carcinoma tissues (%)	Advanced carcinoma tissues (%)
Training samples	100	100	100

Testing samples	99	98	100
